# MENA Delphi consensus on iron deficiency anemia in infants and young children: screening, management, and prevention strategies

**DOI:** 10.3389/fnut.2026.1835042

**Published:** 2026-08-07

**Authors:** Yasmin El Gendy, Eslam El Baroudy, Adlette Inati, Ahmad Al Hammadi, Ali Almehaidib, Ahmad El Beleidy, Ashraf Othman Saleh Sayed, Fatima Ahmed El Haddad, Fawaz El Refaae, Furat Khreshan, Nadine Yazbeck, Peter Noun, Yasser Wali, Ahmed Maged Abdelmawla, Rawane Moufarrej, Naglaa M. Kamal

**Affiliations:** 1 Faculty of Medicine, Ain Shams University, Cairo, Egypt; 2 Faculty of Medicine, Suez University, Suez, Egypt; 3 Department of Health/UAE, "KHMC" Affiliated New York-Presbyterian Children Hospital-Columbia University, Abu Dhabi, United Arab Emirates; 4 Faculty of Medicine and Medical Sciences, and NINI Hospital, University of Balamand, Tripoli, Lebanon; 5 Sidra Medicine, Doha, Qatar; 6 Department of Pediatrics, King Faisal Specialist Hospital & Research Centre, Riyadh, Saudi Arabia; 7 Department of Pediatrics, Faculty of Medicine, Cairo University, Cairo, Egypt; 8 Dar Al Shifa Hospital, Hawally, Kuwait; 9 Faculty of Medicine, Minia University, Minya, Egypt; 10 Government Hospitals, Kingdom of Bahrain, Manama, Bahrain; 11 Pediatric GI Unit, Department of Pediatrics, Al-Adan Hospital, Ministry of Health, Kuwait City, Kuwait; 12 Pediatrics and Early Intervention Center, Amman, Jordan; 13 Department of Pediatrics and Adolescent Medicine, American University of Beirut Medical Center, Beirut, Lebanon; 14 Department of Pediatrics, Hematology Oncology Unit, Saint George Hospital University Medical Center, Saint George University, Beirut, Lebanon; 15 Department of Child Health, Sultan Qaboos University, College of Medicine and Health Sciences, Muscat, Oman; 16 Danone Specialized Nutrition, MENA, Beirut, Lebanon; 17 Kasralainy Faculty of Medicine, Cairo University, Cairo, Egypt

**Keywords:** consensus development conference, dietary, food, infants, iron, iron deficiency anemia, iron rich nutrition, management

## Abstract

**Introduction:**

Iron deficiency anemia (IDA) is one of the most prevalent and consequential nutritional disorders affecting infants and young children globally, with profound implications for neuro development and long-term health. Despite its well-documented risks, IDA remains under diagnosed and under treated, particularly in the Middle East and North Africa (MENA) region, where prevalence ranges from 23.2% in the UAE to 72% in Jordan. To address gaps in screening, diagnosis, and management, a multidisciplinary panel of 14 pediatric experts from nine MENA countries convened to develop evidence-based consensus recommendations.

**Methods:**

The methodology included a structured literature review, virtual consensus meetings, and iterative Delphi-style voting on 32 evidence-based statements across five domains: (1) prevalence and burden, (2) screening and diagnosis, (3) nutritional prevention, (4) treatment, and (5) public health strategies.

**Results:**

Key recommendations included universal screening at 9–12 months using hemoglobin, CRP, and ferritin; first-line oral iron therapy (3–6 mg/kg/day of elemental iron for 3–6 months); and avoidance of cow’s milk before 12 months. The panel also developed a comprehensive algorithm to standardize diagnosis and management across the region. Major barriers identified were underutilized screening practices (85.71% agreement) and insufficient public awareness (92.86% emphasized tailored campaigns). Excessive and early cow’s milk consumption was considered a key dietary contributor to IDA, with 84.6% agreement on its role due to low iron content and 69.2% agreement on its inhibitory effects on iron absorption and risk of gastrointestinal blood loss.

**Discussion:**

This consensus highlights critical interventions, including iron-rich complementary feeding, caregiver education, and referral for refractory cases. By aligning clinical practice with current evidence and regional realities, these consensus recommendations provide a structured framework to reduce the burden of IDA in MENA through early detection, standardized care, and collaborative public health strategies.

## Introduction

Iron deficiency anemia (IDA) is one of the most common and impactful nutritional deficiencies affecting infants and young children worldwide and can have lasting consequences if not addressed early (1–3). It is essential to distinguish between iron deficiency (ID), defined as a state in which iron stores are depleted but hemoglobin synthesis remains unimpaired, and IDA, in which insufficient iron leads to decreased hemoglobin levels and impaired oxygen transport (4). Anemia itself is broadly characterized by a reduction in red blood cell count, hemoglobin concentration, or hematocrit, with thresholds varying by age and gender (3, 5). In children aged 6–59 months, a hemoglobin level below 11 g/dL is generally accepted as indicative of anemia (3). However, while this World Health Organization threshold serves as a general reference, the diagnostic criteria adopted in this consensus use age-specific cutoffs (<10.5 g/dL for children aged 6–23 months) to better reflect physiological variation in early childhood and align with current best clinical practice (6, 7).

IDA often presents with subtle signs. However, the implications can be profound, particularly for neurodevelopment. Evidence shows that iron-deficient infants, particularly those aged 6–24 months, are at an increased risk of cognitive, motor, social–emotional, and neurophysiologic impairments, all of which are effects that may persist into adolescence and beyond despite adequate treatment (8, 9). For example, children with moderate anemia (Hb ≤ 10 g/dL) in infancy scored lower on cognitive tests at school age (10). The developing brain’s dependence on iron for neurometabolism, myelination, and neurotransmitter function underscores the urgency of prevention and early detection as well as the timely treatment of ID and IDA (9).

Globally, IDA represents a significant public health burden (11, 12). According to the World Health Organization (WHO), anemia affects approximately 40% of children aged 6–59 months (11), with ID estimated to account for approximately half of all anemia cases worldwide, although this proportion varies considerably by region and infection burden (13). ID remains the most common single nutritional cause of anemia in young children, alongside its high prevalence among pregnant women and adolescents (1). In the Middle East and North Africa (MENA) region, the challenge is no less severe. In 2021, the age-standardized prevalence of dietary ID reached 14,368.2 per 100,000 population, with Yemen, Sudan, and Morocco showing the highest national rates (14). Country-specific data further highlight the magnitude of the problem: 44% of Egyptian children were reported to be anemic, with IDA accounting for 96% of cases (15); in Jordan, earlier data reported 72% prevalence of IDA in a prospective cohort of urban infants (16), although more recent nationally representative surveys indicate substantially lower but still concerning rates, with 38% of infants aged 6–11 months identified as anemic and 17% diagnosed with IDA in 2020 (17). Nationally, the prevalence of anemia in preschool children declined from 40.4% in 2007 to 33.9% in 2009 following wheat flour fortification (18). In Saudi Arabia, more than half of the infants (51%) were diagnosed with IDA (19); and in the UAE, the incidence of IDA among infants was estimated at 23.2% (20). In Lebanon, mild and moderate anemia affected 71.8 and 25.4%, respectively, of hospitalized children aged more than 6 months (21).

Beyond its hematologic manifestations, IDA contributes to significant developmental and health consequences, including impaired cognition, weakened immunity, stunted growth, and long-term reductions in productivity. The persistence of IDA in the MENA region reflects multiple interrelated factors: a delayed introduction of iron-rich complementary foods, excessive consumption of cow’s milk, high rates of maternal anemia, and short interpregnancy intervals (22, 23).

Despite the high burden and well-documented consequences of IDA, several challenges hinder its effective management. These challenges include variability in screening practices [e.g., a cross-sectional survey of 147 physicians at a single tertiary center in Riyadh found wide variability in IDA diagnostic and therapeutic approaches, with only 15.6% recommending CBC alone as sufficient for nutritional IDA diagnosis (22). Although this single-center study has limited generalizability, the finding is consistent with international data demonstrating substantial practice variation in IDA evaluation (24)]; nutritional strategies [early introduction and excessive cow’s milk intake (>500 mL/day) and delayed introduction of iron-rich foods are major contributors (23)]; and limited caregiver awareness, inconsistencies in diagnostic thresholds, and lack of access to standardized treatment protocols [substantial variations exist in iron dosing (2–6 mg/kg/day) and formulation preferences among clinicians (25)]. The economic and public health implications of untreated IDA—ranging from increased healthcare costs to reduced educational attainment and productivity—further compound its societal toll (26). For example, chronic ID in infancy was associated with lower secondary school completion rates and poorer emotional health in adulthood (27).

Given these challenges, there is a pressing need for unified, evidence-based guidelines tailored to the specific epidemiological and healthcare contexts of the MENA region. This consensus study brings together experts across the region to address key gaps in the prevention, screening, diagnosis, and management of IDA among infants and young children. Our objectives are to assess regional prevalence, identify high-risk populations, evaluate current practices, and develop harmonized strategies that can guide clinicians and public health stakeholders alike. Through this effort, we aim to improve outcomes for affected children and reduce the long-term burden of IDA in the region.

## Methodology

### Expert panel composition

On 11 April 2025, a virtual consensus meeting was held, with 14 pediatric experts from across the MENA region, including Lebanon, Qatar, Saudi Arabia (KSA), Egypt, Abu Dhabi (UAE), Bahrain, Kuwait, Jordan, and Oman. The panel included a multidisciplinary group of pediatricians, pediatric hematologists, pediatric gastroenterologists, neonatologists, and nutrition specialists, chaired by a Professor of Human Nutrition.

### Meeting objectives

The primary objectives of the meeting were to (1) assess the prevalence and public health significance of IDA among infants and young children across the MENA region, (2) review current screening practices and identify gaps in early IDA detection, particularly in high-risk groups, (3) discuss diagnostic challenges, including variability in thresholds, timing of testing, and availability of tools, (4) evaluate management approaches, including supplementation strategies, dietary interventions, and follow-up protocols, (5) emphasize the need for caregiver education and public health campaigns to improve the awareness of IDA prevention and treatment, and (6) develop regionally relevant consensus statements to standardize screening, diagnosis, and management practices.

### Consensus development process

A structured literature review was conducted across PubMed, Scopus, and WHO databases using the terms *iron deficiency anemia, infants, children, MENA region, screening,* and *treatment.* Studies published in English between 2000 and 2025 were included. Using a structured, three-round Delphi method, the panel developed 32 consensus statements across five major themes: (1) Prevalence and Burden of IDA in the MENA Region (six statements), (2) Screening and Diagnosis of IDA (seven statements), (3) Role of Nutrition in Preventing and Managing IDA (nine statements), (4) Treatment and Management of IDA (seven statements), and (5) Public Awareness and Education on IDA in Infants and Young Children (three statements). No pilot testing was conducted.

In the first round (i.e., the pre-meeting phase), all draft statements were distributed electronically to the panel members for independent and anonymous rating according to a predefined agreement scale. Participants were also invited to provide written comments and suggest specific wording modifications. A consensus threshold was defined *a priori* (≥80% agreement). Statements meeting this threshold were provisionally accepted, whereas those not reaching the predefined level of agreement were identified for further discussion during the consensus meeting.

In the second round (i.e., the consensus meeting phase), all statements were systematically reviewed. Statements that had reached the agreement threshold were briefly confirmed, while those below the threshold were discussed in detail. Panel members were asked to clarify their positions (agreement or disagreement), articulate concerns, and propose precise modifications aimed at improving clarity, scientific accuracy, or acceptability. Revisions were made when appropriate to reflect the collective feedback of the group. The objective of the discussion was to refine statements to better represent shared expert opinion rather than to force agreement.

In the third round (i.e., the post-meeting validation phase), all revised statements were redistributed to the panel for a final round of anonymous rating. Participants were asked to confirm their agreement with the revised wording and to indicate whether the statements accurately reflected the meeting discussions. Consensus was redetermined using the predefined agreement threshold (≥80%). Statements that achieved the required level of agreement after revision were accepted as final consensus statements. Statements that remained below the consensus threshold after discussion and re-rating were classified as non-consensus statements and were not included among the final recommendations. Statements were revised only when modifications were supported by substantive feedback, and no statement was reformulated solely to artificially increase agreement. This structured and iterative approach ensured methodological rigor, transparency, and a clear distinction between consensus and persistent expert disagreement.

### Voting and consensus threshold

Each statement was scored using the following criteria: agree, partially agree, and disagree. A consensus was achieved if ≥80% of panelists voted “agree” on a given statement. Recommendations from numerous global governing authorities, including the WHO, were reviewed and adapted to local practices to establish practical, evidence-based guidelines.

### Final output

The meeting concluded with the development of a regionally adapted algorithm for the screening, diagnosis, and management of IDA in infants and young children. The final consensus statements aim to support early detection, appropriate intervention, and long-term prevention strategies across the MENA region.

This structured methodology ensured a rigorous, evidence-based, and collaborative approach to establishing standardized guidelines for IDA in pediatric populations.

## Results and discussion

Through a structured consensus process, the expert panel reached agreement on 24 statements, which were categorized into five key domains. The voting results, along with the final recommendations, are presented below. This section outlines the panel’s endorsed guidelines, providing clinicians and policymakers with actionable strategies to improve the prevention, detection, and treatment of IDA in pediatric populations (see Table 1).

**Table 1:** The prevalence and burden of IDA in the MENA region.

Statements	Consensus (%)	Evidence Level
Iron deficiency anemia (IDA) is an under-recognized public health concern in the MENA region, especially among infants and toddlers aged under 5, where prevalence can reach up to 50%.	Agree 78.57% Partially agree 14.29% Disagree 7.14%	C
IDA in early childhood is linked to both short- and long-term negative effects on cognitive function, growth, immune health, and overall well-being. Some consequences may be irreversible, emphasizing the need for early intervention.	Agree 85.71% Partially agree 14.29% Disagree 0%	A
IDA in infancy is associated with poorer cognitive function, increased behavioral issues, and impaired social development in early adolescence.	Agree 92.86% Partially agree 7.14% Disagree 0%	B
Key risk factors for IDA include being between 6 and 18 months old, male gender, prematurity and low birth weight (LBW), maternal IDA, low socioeconomic status, early introduction of cow’s milk, prolonged exclusive breastfeeding beyond six months without iron supplementation, and transitioning to cow’s milk or iron-poor foods during weaning.	Agree 92.86% Partially agree 7.14% Disagree 0%	B
IDA during pregnancy increases the risk of adverse perinatal outcomes, such as intrauterine growth restriction, prematurity, low birth weight, and postpartum cognitive and behavioral impairments, as well as IDA in the offspring.	Agree 92.86% Partially agree 7.14% Disagree 0%	A
Adequate and timely maternal iron supplementation is essential for preventing iron deficiency in pregnancy and preventing its harmful effects.	Agree 78.57% Partially agree 21.43% Disagree 0.00%	B

Approximately 79% of the experts agreed that IDA remains a significant yet under-recognized public health challenge in the MENA region, particularly affecting infants and young children younger than 5 years, with prevalence rates reaching up to 50% in some areas (e.g., 23.2% in UAE infants and 43% in Egyptian infants 6–24 months old) (28, 29). The discussion highlighted that maternal IDA rates are alarmingly high due to characteristic regional factors such as high delivery rates and short interpregnancy intervals (30), with the panel noting that although large, socioeconomically disadvantaged families are particularly vulnerable, IDA remains prevalent even among higher socioeconomic groups (31), underscoring the need for universal screening and preventive approaches. The panel emphasized the irreversible consequences of untreated IDA, including cognitive and motor delays, behavioral impairments, growth faltering, and weakened immunity (32), which is supported by evidence that infants with moderate anemia (Hb ≤ 10 g/dL) had lower cognitive scores at school age (10). Key risk factors identified included infants aged 6–18 months, male sex, prematurity, low birth weight, maternal IDA, poor dietary practices (e.g., cow’s milk consumption before 12 months (33), socioeconomic factors, and high *Helicobacter pylori* prevalence and parasitic infections contributing to hidden GI blood loss. A majority of the experts agreed that maternal IDA significantly increases the risks of adverse perinatal outcomes and predisposes offspring to IDA (34), emphasizing the crucial need for standardized antenatal iron supplementation protocols (see Table 2).

**Table 2:** Screening and diagnosis of IDA.

Statements	Consensus (%)	Evidence level
In many areas of the MENA region, IDA screening is underutilized, with many cases diagnosed only after symptoms appear, missing the opportunity for early intervention.	Agree 85.71% Partially agree 14.29% Disagree 0%	C
Routine screening for IDA among infants aged 9-12 months is essential and should be a standard practice rather than relying on symptoms.	Agree 92.86% Partially agree 7.14% Disagree 0%	B
Close monitoring of infants for any risk factors for iron deficiency enables early treatment before anemia develops, preventing associated cognitive, motor, and behavioral effects.	Agree 85.71% Partially agree 14.29% Disagree 0%	C
A comprehensive screening approach should include clinical and physical assessments, laboratory tests, and dietary questionnaires to evaluate iron intake and risk factors.	Agree 92.86% Partially agree 7.14% Disagree 0%	C
IDA diagnosis should be based on hemoglobin levels below 10.5 g/dL in children aged 6-23 months and 11 g/dl in children aged 24-59 months, along with low serum ferritin (below 12 mg/L) and normal C-reactive protein (CRP) measurements, or ferritin below 30 µg/L if inflammation is present. For diagnosing ID with and without anemia in infants and children < 5 years, the WHO criteria for low serum ferritin (<12 µg/L) should be used.	Agree 71.43% Partially agree 28.57% Disagree 0%	B
Healthcare providers should have access to effective, cost-efficient screening tools to support early detection of IDA, especially in high-risk populations.	Agree 85.71% Partially agree 14.29% Disagree 0%	C
All infants aged 9–12 months, may benefit from rapid, non-invasive, office-based hemoglobin testing, along with dietary history, to identify nutritional deficiencies.	Agree 100% Partially agree 0% Disagree 0%	C

The expert panel reached strong consensus that IDA screening remains underutilized across the MENA region, with many cases being identified only after symptomatic presentation, thereby missing critical opportunities for early intervention. Strong consensus was achieved on implementing rapid, non-invasive office-based hemoglobin testing, combined with a dietary history for all infants aged 9–12 months, recognizing this as a practical solution to overcome resource limitations. A majority of the experts agreed that routine universal screening at 9–12 months (35), supported by evidence that CHr (reticulocyte hemoglobin content) < 27.5 pg. is more sensitive than Hb for the early detection of iron deficiency (36)), should be preferred over symptom-dependent diagnosis (which is not widely available). The panel also emphasized the importance of close monitoring of infants with any risk factors to prevent the development of IDA-associated cognitive, motor, and behavioral impairments. Diagnostic approaches generated nuanced discussion, with 71.43% of experts agreed on using WHO hemoglobin cutoffs (<10.5 g/dL for 6–23 months; <11 g/dL for 24–59 months) (37), combined with ferritin levels after adjusting for inflammatory confounders, although 28.57% of experts partially agreed due to concerns about inflammatory confounders in regional populations. The panel strongly supported comprehensive screening that incorporates clinical assessment, laboratory evaluation, dietary iron intake, and dietary history, while advocating for wider availability of cost-effective tools to facilitate early detection, particularly highlighting the value of point-of-care testing in resource-limited settings. A majority of the experts recognized that physicians are aware of IDA but face systemic challenges in its management, including the absence of related screening protocols and inconsistent follow-up; hence, national health ministries should implement universal, non-invasive screening programs and develop region-specific guidelines to address this preventable condition with lifelong consequences (see Table 3).

**Table 3:** Role of nutrition in preventing and managing IDA.

Statements	Consensus (%)	Evidence level
Starting at 6 months of age, all infants and toddlers should consume iron-rich complementary foods, including iron-fortified cereals, legumes, pulses, and, if culturally appropriate, meat, eggs, and poultry.	Agree 78.57% Partially agree 14.29% Disagree 7.14%	A
Young children have high nutritional needs for proper growth and development, yet many infants and toddlers in the MENA region consume iron-poor, and low-diversity diets, increasing their risk of IDA.	Agree 71.43% Partially agree 28.57% Disagree 0%	B
Diets of young children in the MENA region often lack essential micronutrients, including iron, zinc, folic acid, vitamin D, vitamin A, and essential fatty acids (EFAs).	Agree 85.71% Partially agree 14.29% Disagree 0%	B
Cow’s milk contains low levels of iron (0.5 mg/l) making it insufficient to meet the daily iron needs of infants and toddlers.	Agree 78.57% Partially agree 21.43% Disagree 0%	A
Excessive cow’s milk consumption not only fails to provide adequate iron but also inhibits iron absorption and may increase gastrointestinal iron losses, increasing the risk of IDA. Additionally, foods containing phytates and polyphenols should be limited, and public health recommendations should address these dietary risks.	Agree 64.29% Partially agree 35.71% Disagree 0%	A
Exclusive breastfeeding is essential for the first 6 months. However, these infants often require iron supplementation (1–2 mg/kg/day) after 4 months of age.	Agree 71.43% Partially agree 28.57% Disagree 0%	B
In non-breastfed infants, iron-fortified formulas (4–12 mg iron/l) should be given, particularly in populations with low dietary iron intake. In non-breastfed infants, healthcare professionals should promote the use of iron-fortified formulas along with iron-rich foods for at-risk infants and toddlers, to reduce risk of IDA.	Agree 85.71% Partially agree 14.29% Disagree 0%	A
After 12 months of age, providing 300–600 ml of young child formula (YCF) or iron-fortified formula is one of the strategies for reducing IDA in infants and toddlers.	Agree 92.86% Partially agree 7.14% Disagree 0%	C
YCF supplemented with prebiotics (e.g., GOS: FOS) and vitamin C may enhance iron absorption, further supporting its role in IDA prevention.	Agree 100% Partially agree 0% Disagree 0%	B

While most experts supported introducing iron-rich complementary foods (fortified cereals, legumes, meat, poultry, and eggs) at 6 months of age (38), they noted regional variations in food availability and cultural practices. The panel highlighted that many children in the MENA region consume diets that are deficient in multiple micronutrients, including iron, zinc, folate, and vitamin D, thereby exacerbating the risk of IDA (39). A key concern was excessive cow’s milk consumption (≥500 mL/day), with 78.57% of experts agreeing that its low iron content (0.5 mg/L) fails to meet the nutritional needs, while 64.29% of experts emphasized its inhibitory effects on iron absorption and its potential to cause gastrointestinal blood loss (23). Moreover, 64.29% of the experts agreeing on limiting phytate- and polyphenol-rich foods that impair iron absorption (40). Regarding infant feeding, 71.43% of experts agreed that exclusively breastfed infants require iron supplementation (1–2 mg/kg/day) starting at 4 months (3), while non-breastfed infants should receive iron-fortified formula (4–12 mg/L) (41). Strong consensus was reached on the benefits of YCF enriched with prebiotics (GOS: FOS) and vitamin C to preserve iron status (42), supported by *in vitro* evidence that prebiotics and ascorbic acid significantly improve iron bioavailability (43). Post-12 months, 92.86% endorsed 300–600 mL/day of YCF or iron-fortified formula as an effective preventive measure (44) (see Table 4).

**Table 4:** Treatment and management of IDA.

Statements	Consensus (%)	Evidence Level
Families of children with IDA should receive counseling on limiting daily cows’ milk intake and increasing iron-rich foods, particularly those paired with vitamin C and prebiotics to enhance absorption.	Agree 85.71% Partially agree 7.14% Disagree 7.14%	B
Effective management of iron deficiency includes iron supplementation, dietary modifications to increase iron intake, and monitoring of the response to treatment.	Agree 100% Partially agree 0% Disagree 0%	A
Oral iron supplementation is the standard first-line treatment for IDA in infants and toddlers. Iron should be administered in combination with heme iron- and vitamin C-rich foods to optimize its absorption.	Agree 92.86% Partially agree 7.14% Disagree 0%	A
The recommended therapeutic dose of oral elemental iron is 3–6 mg/kg/day for three to six months, with appropriate follow-up to ensure complete replenishment of body iron stores.	Agree 92.86% Partially agree 7.14% Disagree 0%	B
If oral iron supplementation fails despite good compliance and optimal dosing, further evaluation and referral to a specialist is recommended.	Agree 85.71% Partially agree 14.29% Disagree 0%	C
Poor adherence to oral iron supplementation is a major challenge, due to frequent gastrointestinal side effects and parental concerns. Educational and support strategies are often needed to improve adherence.	Agree 92.86% Partially agree 7.14% Disagree 0%	B
Long-term dietary modifications and the adoption of healthy eating habits are essential to maintain optimal body iron stores.	Agree 100% Partially agree 0% Disagree 0%	B

The expert panel reached unanimous consensus that effective IDA management requires a multifaceted approach, combining iron supplementation, dietary modifications, and close monitoring of treatment response. Oral iron supplementation was strongly endorsed as the first-line therapy (45), with a recommended dose of 3–6 mg/kg/day of elemental iron for 3–6 months to ensure complete replenishment of iron store. To optimize absorption, the panel emphasized administering oral iron together with heme iron- and vitamin C-rich foods, citing evidence that ascorbic acid reduces the inhibitory effects of phytates and tannins (46) while also recommending providing family counseling to limit cow’s milk intake and promote iron-rich diets enhanced with vitamin C and prebiotics (47). Despite treatment efficacy, poor adherence remains a major challenge, often due to gastrointestinal side effects or parental concerns, necessitating tailored education and support strategies. In cases of treatment failure despite compliance, further evaluation and referral to a specialist are recommended. Finally, the panel unanimously agreed that long-term dietary changes and healthy eating habits are crucial for sustained iron sufficiency, highlighting the need for ongoing nutritional guidance to prevent recurrence (see Table 5).

**Table 5:** Public awareness and education on IDA in infants and young children.

Statements	Consensus (%)	Evidence Level
Limited awareness among both parents and healthcare professionals contributes to the high prevalence of IDA in the MENA region.	Agree 100% Partially agree 0% Disagree 0%	C
Healthcare professionals should receive structured training on IDA prevention, screening, and management.	Agree 100% Partially agree 0% Disagree 0%	C
Educational programs and public health campaigns should emphasize the importance of iron-rich complementary feeding, adherence to iron supplementation during IDA treatment, and age-appropriate feeding practices, including limiting excessive cow’s milk consumption during early childhood.	Agree 92.86% Partially agree 7.14% Disagree 0%	C

The expert panel unanimously agreed that limited awareness among parents, caregivers, and healthcare professionals significantly contributes to the high prevalence of IDA in the MENA region. To address this gap, they strongly advocated for structured training programs for healthcare providers, focusing on prevention, early screening, and effective management of IDA. These programs should emphasize evidence-based practices, including the timely introduction of iron-rich complementary foods, appropriate adherence to iron supplementation, and the risks associated with excessive cow’s milk consumption in young children. Additionally, the panel endorsed targeted public health campaigns to improve caregiver knowledge on screening, iron-rich diets, optimal feeding practices, and strategies to enhance iron absorption. Such initiatives are crucial for empowering families and healthcare providers to combat IDA more effectively across the region.

## Algorithm

**Figure 1:**
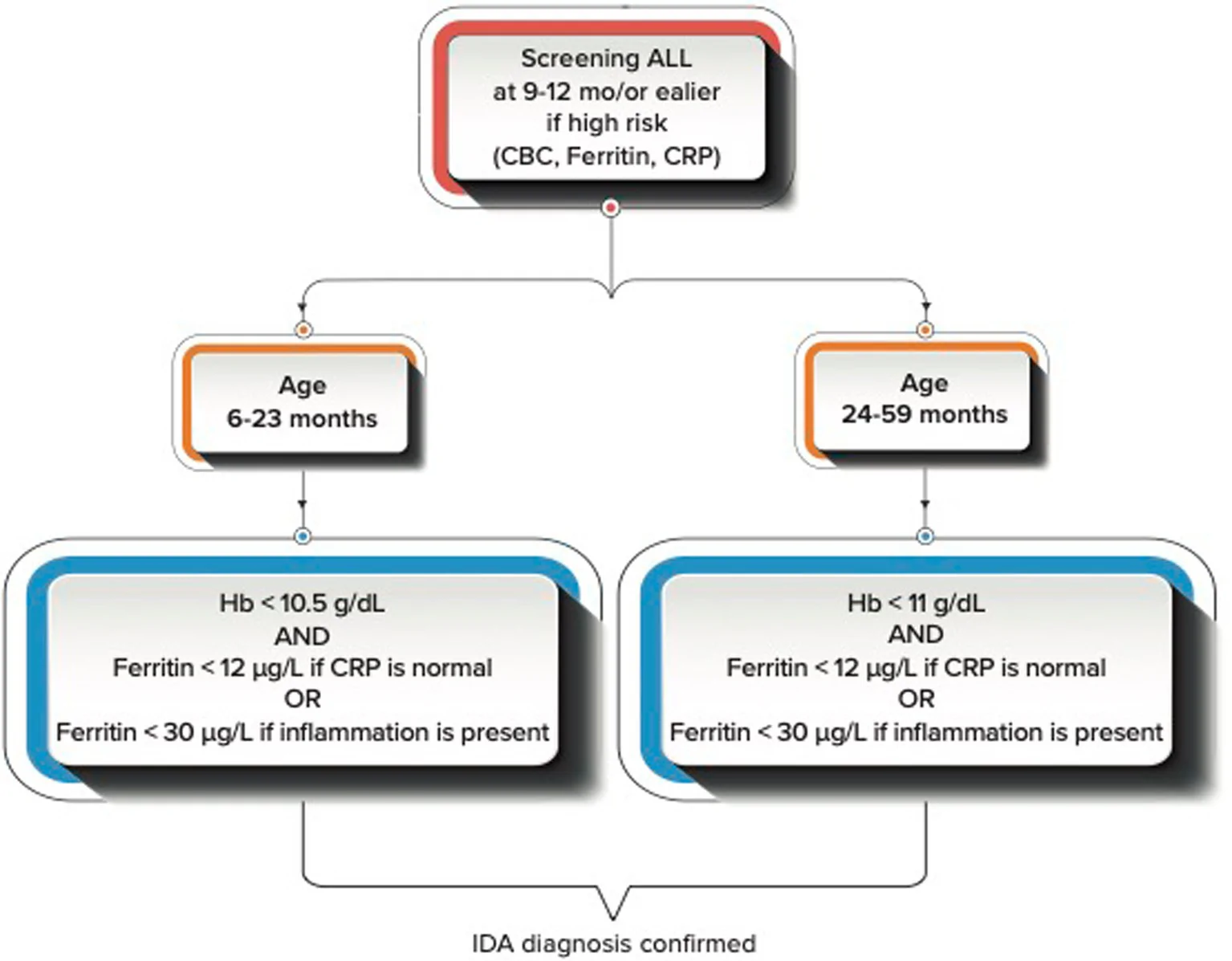
Diagnostic algorithm for IDA in infants and young children (ages 6–59 months).

**Figure 2:**
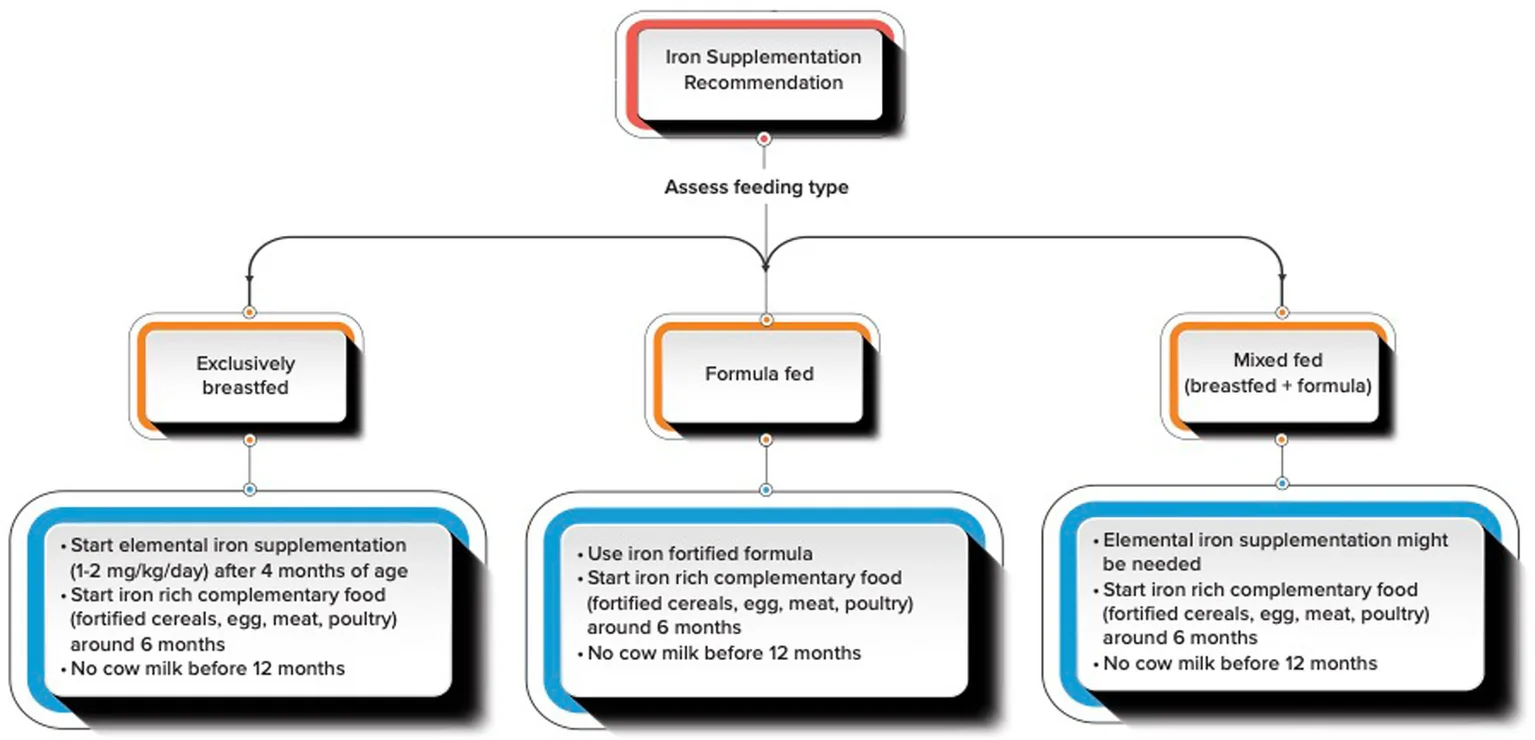
Treatment and monitoring protocol for confirmed IDA diagnosis.

**Figure 3:**
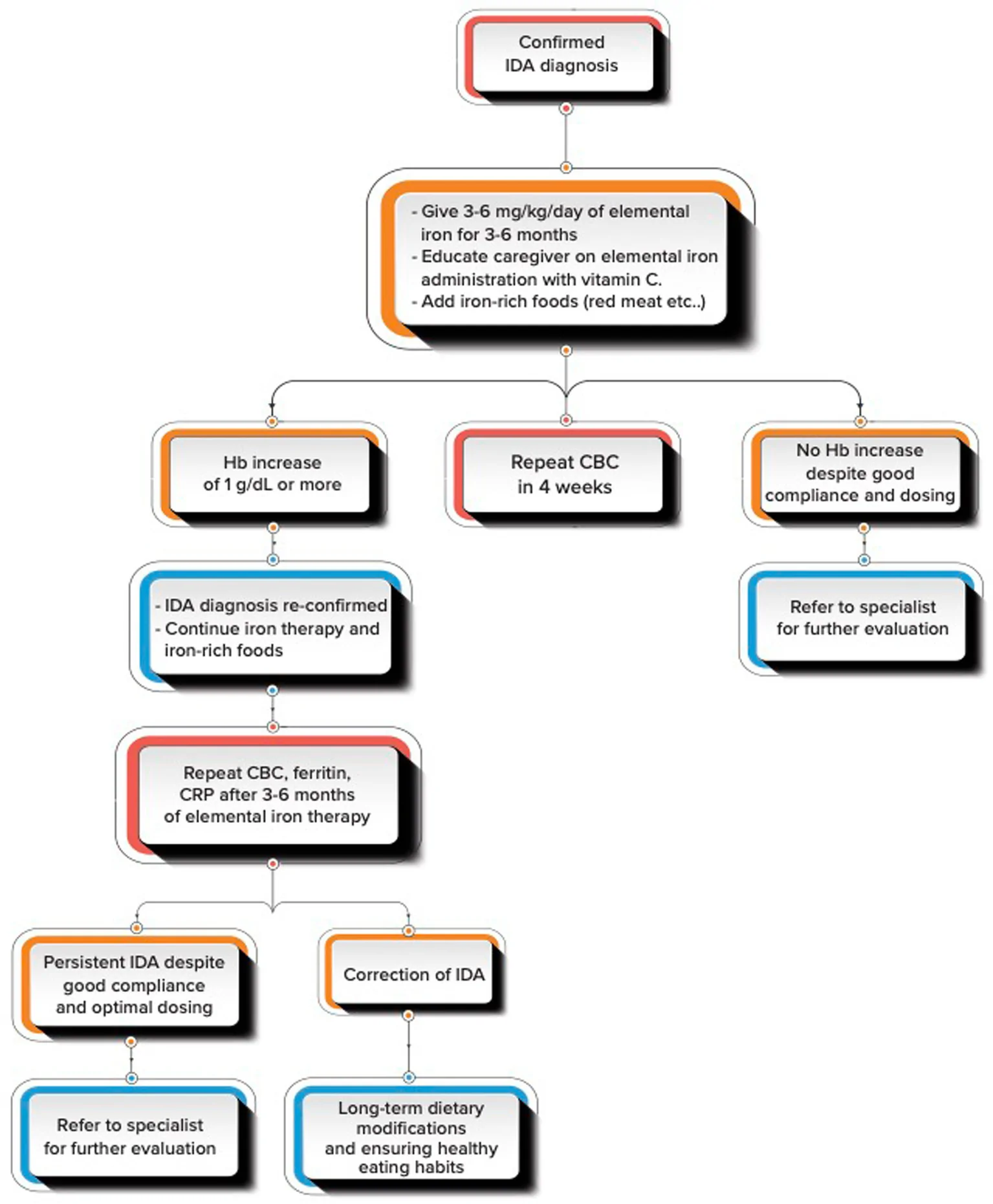
Iron supplementation and feeding recommendations by infant feeding type.

Following extensive discussion and voting on consensus statements, the expert panel developed a comprehensive algorithm to standardize the diagnosis and management of IDA in infants and young children across the MENA region. The algorithm begins with universal screening at 9–12 months (or earlier for high-risk infants), using CBC, ferritin, and CRP to confirm IDA based on age-specific hemoglobin cutoffs (<10.5 g/dL for infants aged 6–23 months; <11 g/dL for infants aged 24–59 months) and ferritin levels adjusted for inflammation (Figure 1). For confirmed IDA, first-line treatment involves oral elemental iron (3–6 mg/kg/day for 3–6 months) paired with vitamin C- and iron-rich foods, followed by monitoring of hemoglobin response after 4 weeks of therapy. If no improvement in hemoglobin occurs despite therapy adherence, specialist referral is recommended (Figure 2). The algorithm also provides feeding-specific iron supplementation guidance: Exclusively breastfed infants should receive iron (1–2 mg/kg/day) from 4 months, while formula-fed infants should use iron-fortified formula (4–12 mg/L), with all groups transitioning to iron-rich complementary foods at 6 months and to YCF after 1 year, while avoiding cow’s milk before 12 months. Long-term dietary modifications and follow-up testing ensure sustained iron sufficiency (Figure 3). This structured approach aims to bridge gaps in early detection, treatment adherence, and dietary practices to reduce IDA’s regional burden.

## Limitations

This expert consensus has several limitations to consider. First, the relatively small number of participating experts (*n* = 14) may not capture the full diversity of clinical perspectives across the MENA region, though all were carefully selected as leading pediatric specialists in their respective countries. Second, while the recommendations are evidence-based, the scarcity of comprehensive regional studies on IDA in children necessitated some generalized statements where local data was unavailable. Third, practical implementation may face challenges due to variations in healthcare infrastructure and resources and inconsistent screening, diagnosis, and treatment, hindering effective public health across different MENA countries. Additionally, the consensus process, while rigorous, relied on expert opinion when definitive data was lacking. The involvement of industry funding and participation of sponsor-affiliated coauthors may be perceived as a potential source of bias despite their non-voting role. Despite these limitations, the document provides valuable, clinically oriented guidance tailored to the region’s needs, and serves as an important foundation for future research and policy development in pediatric IDA management.

## Conclusion

IDA remains a pressing yet underprioritized public health challenge in the MENA region, with significant consequences for infant and child development. This expert consensus highlights the critical gaps in current practice, including inconsistent screening, delayed diagnosis, and suboptimal treatment adherence, that contribute to IDA’s persistent burden. The panel unequivocally endorsed standardized screening among infants aged between 9 and 12 months, regionally adapted diagnostic criteria, and evidence-based iron supplementation protocols to ensure timely intervention. Equally vital are strategies to empower caregivers through targeted education on iron-rich nutrition and the risks of excessive and early cow’s milk consumption. The consensus calls for urgent, collaborative action among healthcare providers, policymakers, and public health authorities to implement regional and national IDA guidelines, improve clinician training, and launch awareness campaigns. By prioritizing prevention, early detection, harmonizing management approaches, and addressing systemic barriers, the MENA region can mitigate the long-term developmental and economic impacts of IDA. These recommendations provide an actionable framework to safeguard the health and developmental potential of infants and young children across the MENA region.

## Data Availability

The original contributions presented in the study are included in the article/supplementary material, further inquiries can be directed to the corresponding author.
